# Lightweight Gearbox Fault Diagnosis Under High Noise Based on Improved Multi-Scale Depthwise Separable Convolution and Efficient Channel Attention

**DOI:** 10.3390/s26041196

**Published:** 2026-02-12

**Authors:** Xiubin Liu, Wei Li, Haoming Li, Yong Zhu, Ramesh K. Agarwal

**Affiliations:** 1 National Research Center of Pumps, Jiangsu University, Zhenjiang 212013, China; 2222311014@stmail.ujs.edu.cn (X.L.); lhm17826076819@163.com (H.L.);; 2 Institute of Fluid Engineering Equipment Technology, Jiangsu University, Zhenjiang 212009, China; 3 Department of Mechanical Engineering & Materials Science, Washington University in St. Louis, St. Louis, MO 63130, USA

**Keywords:** gearbox fault diagnosis, multi-scale feature extraction, efficient channel attention, lightweight model, noise robustness

## Abstract

Gearbox fault diagnosis under strong-noise conditions remains challenging due to the difficulty of extracting weak fault-related features from noise-dominated vibration signals, inefficient modeling of multi-scale impulsive characteristics under limited computational resources, and degraded diagnostic stability across varying noise levels. To address these issues, this paper proposes a lightweight fault diagnosis model (DSMC-ECA) that integrates an improved multi-scale depthwise separable convolution scheme with efficient channel attention. The proposed model adopts a dual-branch parallel feature extraction architecture: the SMC branch captures local fine-grained impulsive features, while the SMDC branch expands the receptive field via multi-scale separable dilated convolutions to model long-range dependencies. Meanwhile, ECA is embedded into the multi-scale features for channel-wise recalibration, highlighting fault-relevant discriminative information and suppressing noise disturbances. The model contains only 0.204 M parameters and requires 10.037 M FLOPs, achieving a favorable trade-off between performance and efficiency. Experimental results on the XJTU and SEU datasets demonstrate that DSMC-ECA consistently outperforms baseline methods across a wide range of signal-to-noise ratios (from −6 dB to noise-free conditions). Notably, under the most challenging −6 dB setting, it achieves the highest average diagnostic accuracies of 95.11% (XJTU) and 86.84% (SEU).

## 1. Introduction

Gearboxes are key motion components in rotating machinery, enabling power transmission and speed regulation, and they are widely deployed in critical industrial equipment such as wind turbines. The gearbox health condition strongly influences overall operating performance and safety [1]. For fluid machinery (e.g., pumps), start-up and shutdown transients can induce pronounced variations in energy conversion and flow structures, which in turn affect efficiency and operational reliability; related studies—covering transient modeling, reduced-order analysis, and investigations/prediction of unsteady flow mechanisms—further underline the importance of safe and dependable operation of such essential equipment [2,3,4,5,6]. Under long-term high-load service and harsh environments, gearboxes frequently develop typical defects such as gear pitting, wear, cracking, and bearing damage. These degradations often exhibit weak early signatures, fast evolution, and strong coupling between fault modes; if not identified promptly, they may lead to severe performance deterioration and even safety accidents. Therefore, accurate gearbox fault detection and diagnosis is crucial for enabling predictive maintenance and supporting safe, stable operation [7,8,9].

Conventional gearbox diagnosis commonly relies on expert knowledge and prior assumptions. Consequently, choices of parameters, feature construction, and operating-condition transfer can be highly subjective and experience-driven; under realistic industrial noise and pronounced condition fluctuations, the generalization and robustness of such workflows can be limited [10]. Similar difficulties have also been reported in online monitoring of other rotating equipment operating under variable loads and variable conditions, where condition disturbances can make diagnostic features unstable—highlighting the broader need for robust diagnosis in complex operating environments [11]. To improve objectivity and accuracy, vibration-based condition monitoring has been extensively adopted, forming representative pipelines centered on time-domain, frequency-domain, and time–frequency representations. Common signal decomposition and time–frequency analysis tools include empirical mode decomposition (EMD) [12], wavelet transform (WT) [13], short-time Fourier transform (STFT) [14], fast Fourier transform (FFT) [15], and adaptive-window Gabor transform methods for variable operating conditions [16]. In addition, for transmission systems with notable speed fluctuation, order analysis and other speed-synchronous spectral techniques are frequently used to mitigate the distortion of spectral features caused by nonstationary rotational speed, thereby improving fault separability under variable-speed regimes [17]. Meanwhile, variational mode decomposition (VMD) and its adaptive variants have also been employed for component extraction and enhancement of fault-related features, providing support for vibration diagnosis in complicated scenarios [18]. For example, Wang et al. combined VMD-related strategies with wavelet analysis and introduced entropy-based indicators to improve discriminability under heavy noise [19]; Yu et al. utilized STFT-based time–frequency representations together with CNNs for gearbox fault identification, achieving improved accuracy to some extent under complex conditions [20]. Nevertheless, many traditional procedures still depend on intensive pre-processing and hand-crafted feature design. When facing strong noise, nonstationarity, and multi-scale coupled vibrations, these approaches often suffer from insufficient feature robustness and high engineering implementation cost; survey studies further emphasize the value of end-to-end robust representation learning [21].

In recent years, deep learning—owing to its end-to-end representation learning capability—has become a major direction for intelligent fault diagnosis of rotating machinery. Convolutional neural networks (CNNs), benefiting from local receptive fields and weight sharing, have shown strong feature extraction performance across various diagnostic tasks. Jing et al. applied CNNs to gearbox diagnosis and reported that frequency-domain inputs (e.g., spectra) can be advantageous for learning fault-sensitive patterns [22]. Eren et al. proposed a compact adaptive 1D-CNN that learns high-resolution features directly from raw vibration signals, achieving good performance with reduced model complexity [23]. Guo et al. fed continuous wavelet transform scalograms into CNNs and validated the method on an experimental platform [24]. Despite these advances, practical industrial applications are often affected by noise contamination and distribution shifts, which may still degrade generalization. To enhance noise tolerance and cross-domain adaptability, Zhang et al. developed a deep model with anti-noise and domain adaptation capability and demonstrated more robust diagnosis on raw vibration signals [25]. In addition, Liu et al. combined a 1D convolutional autoencoder with a 1D-CNN to alleviate noise interference and improve recognition stability under noisy environments [26].

In real industrial settings, fault samples are often scarce and imbalanced, and vibration measurements can be jointly influenced by load variation and strong noise. A single CNN may be limited in modeling long-range temporal dependencies, whereas recurrent neural networks (RNNs) and their variants are more suitable for sequence modeling. Chen et al. proposed a hybrid CNN–LSTM framework to integrate end-to-end feature learning with temporal dependency modeling, although its parameter scale and training cost can be relatively high [27]. Meanwhile, more powerful temporal architectures (e.g., bidirectional convolutional LSTM networks) have also been explored for planetary gearbox fault diagnosis; however, their deployment cost in resource-constrained scenarios still requires careful consideration [28].

To balance diagnostic accuracy and computational cost, lightweight network design has attracted increasing attention. Studies on CNN architectures and optimization suggest that simply deepening networks or enlarging kernel sizes may improve representational capacity, yet often come with substantially higher computation costs and reduced training stability [29,30,31]. In this context, depthwise separable convolution has become popular because it can markedly reduce parameters and FLOPs while preserving strong feature extraction capability, and it has formed a mature paradigm in lightweight visual networks [32]. In the fault diagnosis domain, Ma et al. introduced depthwise separable convolution into a lightweight deep residual CNN and achieved an effective trade-off between accuracy and complexity [33].

Attention mechanisms also provide a useful way to emphasize informative fault cues while suppressing irrelevant disturbances. The squeeze-and-excitation (SE) module strengthens feature selection via channel re-calibration [34], and CBAM further combines channel and spatial attention to improve feature focusing [35]. ECA achieves efficient channel attention through local cross-channel interaction, enabling channel weighting without fully connected layers [36]. For rotating machinery diagnosis, Wang et al. integrated multi-scale CNNs with GRU to jointly model multi-scale feature extraction and temporal dependencies [37]. Shen et al. combined separable multi-scale convolution with broadcast self-attention under lightweight constraints to further improve diagnostic performance [38].

Meanwhile, to alleviate the distribution mismatch between different operating conditions and improve model generalization, transfer learning and domain adaptation methods have been increasingly applied in gearbox fault diagnosis. By mining and leveraging prior knowledge from related operating conditions or similar equipment, these approaches can effectively reduce the reliance on labeled samples in the target condition and enhance diagnostic robustness across varying operating conditions, loads, or noise levels. Several solutions have been proposed in previous studies: the Integrated-Dispersion Manifold Distance (IDMD) captures both global distribution shifts and local manifold differences between the source and target domains to achieve more accurate feature alignment under complex conditions [39]; Federated Transfer Learning (FTL) enables cross-domain knowledge sharing while preserving data privacy, which is particularly suitable for industrial scenarios with data islands [40]; conditional distribution-guided adversarial transfer networks, adaptive fused domain-cycling variational generative adversarial networks, and dynamic collaborative adversarial domain adaptation networks [41,42,43] employ adversarial learning and generative feature alignment to improve cross-condition diagnostic stability and accuracy.

Despite achieving certain improvements in feature extraction and cross-domain generalization, these methods still have practical limitations. They often rely on relatively complex network architectures, additional temporal modeling modules, or attention mechanisms with large parameter overheads, which increase computational cost and training complexity, limiting their deployment in resource-constrained industrial settings. More specifically, current gearbox fault diagnosis under strong-noise conditions still faces the following three key challenges:

1. Feature extraction under strong-noise conditions: In industrial environments, gearbox vibration signals are often accompanied by high background noise, making it difficult to capture key fault information. Existing lightweight or deep learning methods frequently suffer from feature saturation in noisy conditions, where noise dominates the model’s learning and weakens critical fault features.
2. Efficient modeling of multi-scale features: Gearbox fault signals typically contain impact and vibration patterns at different time scales. Effectively capturing these multi-scale features is crucial for improving diagnostic accuracy. However, multi-scale convolutions or attention mechanisms are often inefficient under limited computational resources, which may increase model complexity and hinder practical deployment.
3. Stability and generalization across noise levels and varying operating conditions: Gearboxes operate under varying loads, speeds, and environmental noise levels, leading to distribution shifts that can degrade model performance in new conditions. Existing methods employ transfer learning or adversarial domain adaptation to improve generalization, but they usually rely on complex networks and training strategies, which increase deployment difficulty.

Based on these observations, this paper develops a lightweight gearbox fault diagnosis model by combining an improved multi-scale separable convolution design with Efficient Channel Attention (ECA). The contributions of this work are listed below:

1. Improved multi-scale depthwise separable convolution design: We propose a dual-branch multi-scale depthwise separable convolution module. By reorganizing the order of depthwise and pointwise convolutions and introducing dilated convolution in one branch, the module enables joint modeling of local impulsive components and cross-scale correlated features with reduced parameters and computational cost.
2. Lightweight channel-attention-driven feature recalibration: By incorporating an Efficient Channel Attention (ECA) mechanism, channel-wise feature reweighting is achieved at a very low computational expense. This helps produce clearer fault-related representations with less noise interference, thereby substantially improving diagnostic accuracy and model robustness.
3. Efficiency and robustness under noise: The proposed dual-branch separable multi-scale structure substantially reduces parameters and computational cost, while maintaining stable and accurate diagnosis across multiple noise levels, with clear advantages in severe-noise regimes.

## 2. Related Theories and Methods

### 2.1. Multi-Scale Convolutional Neural Network (MSCNN)

A multi-scale convolutional neural network (MSCNN) is a deep learning framework that employs parallel multi-branch convolutions to construct receptive fields of different sizes, thereby enabling joint modeling of multi-scale features. In conventional CNNs, a single layer typically uses convolution kernels with a fixed scale and is therefore mainly sensitive to patterns at one specific resolution. However, gearbox vibration signals usually contain fault signatures across multiple frequency bands, including high-frequency impulsive components and low-frequency modulation information. Consequently, multi-scale feature extraction helps characterize both local details and global structural patterns of complex signals more comprehensively.

**Figure 1:**
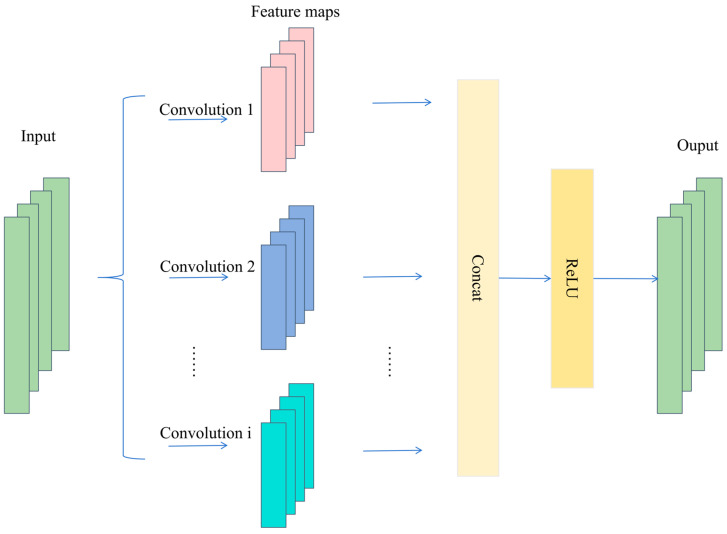
Structure of the MSCNN.

As displayed in Figure 1, the MSCNN mainly consists of a multi-scale convolution layer, a feature fusion layer, and nonlinear activation layers. Specifically, the multi-scale convolution layer applies multiple convolution kernels with different sizes to the input in parallel, so as to capture feature patterns at different scales. The convolution operation at the *i*-th scale can be expressed as:

(1) $$Y_{i}=W_{i}*X+b_{i}$$

where $W_{i}$ denotes the convolution kernel at the *i*-th scale, $b_{i}$ is the bias term, X represents the feature map extracted at this scale and $Y_{i}$ denotes the feature extracted at the corresponding scale. Because different kernel sizes correspond to different receptive fields, smaller kernels focus more on fine-grained local details, whereas larger kernels are more effective in capturing broader, more global characteristics.

Then, the outputs of all branches are concatenated along the channel dimension (Concat) to form a unified multi-scale representation:

(2) $$F=Concat\left(Y_{1},Y_{2},\ldots ,Y_{n}\right)$$

where $Y_{i}$ denotes the feature map from the *i*-th scale, and F is the fused multi-scale feature representation. This fusion strategy preserves the diversity across scales while leveraging complementary information among multi-scale features.

Finally, a nonlinear activation function is introduced to enhance the representational capacity for complex patterns. After fusion, a ReLU activation is applied to obtain the output feature:

(3) Y=ReLUF=max0,F

where Y denotes the final output feature of the MSCNN module.

### 2.2. Improved Multi-Scale Separable Convolution

In rotating machinery fault diagnosis, vibration signals usually exhibit complex characteristics, including multiscale structures, non-stationarity, and strong interference coupling. To effectively represent various impulsive and modulation components embedded in such signals, multiscale convolutional structures are commonly employed for feature extraction. However, conventional multiscale convolution methods are typically implemented using multiple parallel branches, where each branch adopts standard convolution operations. As the number of branches or channels increases, the parameter count and computational cost grow rapidly, which limits their applicability in resource-constrained deployment scenarios.

Depthwise separable convolution divides a conventional convolution into a depthwise convolution followed by a pointwise convolution, allowing it to be integrated into multiscale architectures. This decomposition significantly reduces the number of convolution parameters and computational overhead while preserving effective feature extraction capability.

Based on this idea, Shen et al. [38] proposed a separable multiscale convolution (SMC) module in LiConvFormer. Unlike approaches that repeatedly perform pointwise convolution within each scale branch, the SMC module adopts a strategy of shared pointwise convolution prior to multiscale processing. Specifically, a shared 1 × 1 convolution is first applied to the input feature map to achieve cross-channel information interaction. Subsequently, the transformed features are processed in parallel by multiple scale branches using different convolution kernel sizes to extract local multiscale information. The outputs from all branches are then concatenated along the channel dimension and further processed by normalization and nonlinear activation to produce the final output of the SMC module. Since pointwise convolution is performed only once before the multiscale branches, SMC effectively avoids redundant channel fusion operations within individual branches, thereby reducing unnecessary computational overhead. As a result, the SMC module is capable of efficiently capturing effective multiscale local features while satisfying lightweight model requirements.

**Figure 2:**
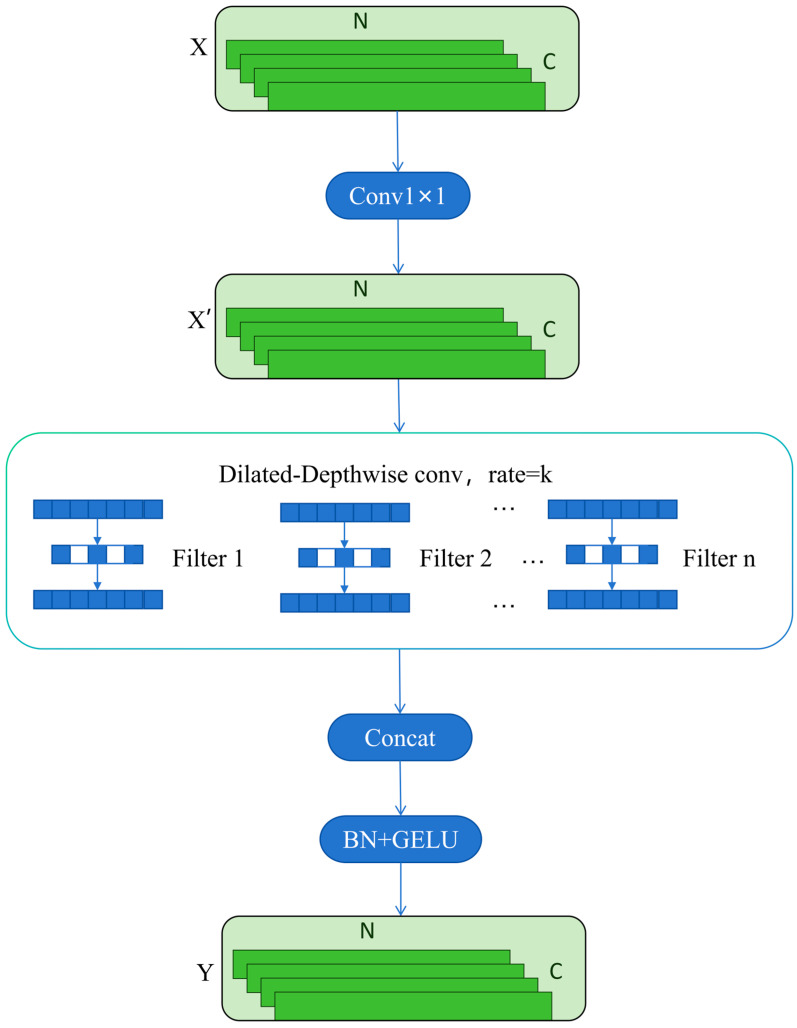
Architecture of the SMDC module.

Although SMC is advantageous in terms of parameter efficiency, gearbox vibration signals often contain long-range dependencies, such as cross-period correlations and broadband modulations. Relying solely on local convolutions with limited kernel sizes may yield an insufficient effective receptive field, which can restrict the modeling of long-range discriminative information. To further enlarge receptive field coverage and strengthen long-range dependency representation, this paper incorporates a dilation mechanism into the SMC framework and proposes a separable multi-scale dilated convolution (SMDC) module, as illustrated in Figure 2.

The overall pipeline of SMDC is similar to that of SMC. The key difference is that the standard depthwise convolutions are replaced with dilated depthwise convolutions, and different dilation rates d are assigned to different scale branches. In this way, the receptive field can be expanded without increasing the number of parameters, enabling collaborative modeling of local impulsive details and cross-period modulation structures. For a 1D dilated convolution with kernel size k, the effective kernel size is given by:

(4) $$k^{’}=k+\left(k-1\right)\times \left(d-1\right)$$

where $k^{’}$ denotes the effective kernel size, *k* is the original kernel size, and *d* represents the dilation rate.

By properly selecting dilation factors across branches, the SMDC module can capture both fine-grained local details and long-range dependency information within a multi-scale feature space, thereby better accommodating the non-stationary behavior and multi-scale feature distribution typically observed in gearbox fault signals.

### 2.3. Efficient Channel Attention (ECA)

Efficient Channel Attention (ECA) is a lightweight channel-attention mechanism designed to enhance a network’s ability to emphasize informative channel features with nearly no additional parameters. Unlike the classical squeeze-and-excitation (SE) module, ECA removes the fully connected layers and the channel reduction operation. Instead, it applies a 1D convolution directly to the channel descriptor obtained by global average pooling, enabling local cross-channel interaction and effectively avoiding information loss caused by dimensionality reduction.

**Figure 3:**
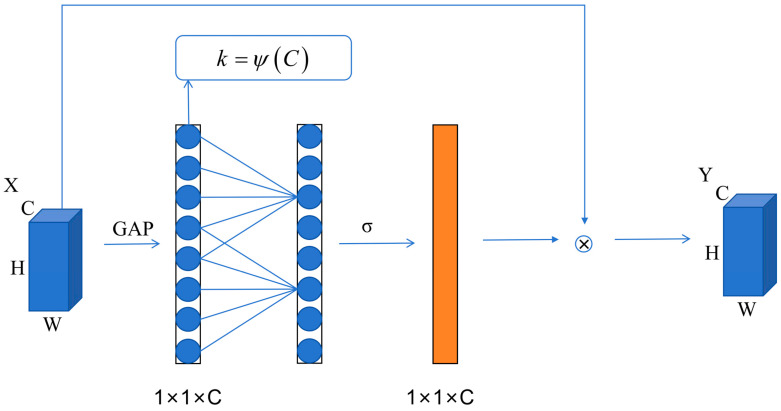
Structure of the ECA module.

The overall architecture of ECA is shown in Figure 3, and the computation can be summarized as follows.

First, global average pooling (GAP) is performed to squeeze the spatial dimensions of each channel, producing a channel descriptor that reflects the global semantic response of each channel $Z_{c}$:

(5) $$Z_{c}=\frac{1}{H\times W}\sum_{i=1}^{H}\sum_{j=1}^{W}X_{i,j}$$

where *H* and *W* denote the spatial size of the feature map, and $X_{i,j}$ represents the value at spatial location (*i*,*j*) in the c-th channel.

To capture inter-channel dependencies, ECA introduces a 1D convolution with kernel size *k* (without nonlinear activation and without bias). By sliding the convolution window along the channel dimension, local cross-channel information interaction is achieved:

(6) $$a=\sigma \left(\mathrm{Conv}1D_{k}\left(Z\right)\right)$$

where σ(·) is the Sigmoid activation function, Conv1D(·) denotes the bias-free 1D convolution and a represents the computed channel-wise attention weights.

To adapt the receptive range of channel-dependency modeling to feature maps of different channel widths, ECA further proposes that the kernel size *k* should be self-adaptively determined by the channel number *C*:

(7) $$k=\psi \left(C\right)={\left|\frac{\log_{2}\left(C\right)}{\gamma }+b\right|}_{odd}$$

where γ and *b* are hyperparameters controlling the kernel size (recommended values are γ = 2 and *b* = 1 in the original paper), and ${\left|\cdot \right|}_{odd}$ denotes rounding up to the nearest odd integer to ensure a symmetric kernel with a well-defined center. This adaptive strategy helps avoid overfitting when the channel number is small, while enabling richer dependency modeling as *C* increases, thereby balancing lightweight design and representational capacity.

Finally, the original features are activated and weighted, and the resulting attention weights are applied to the original input feature map:

(8) Y=a⊗X

where Y denotes the final output feature, ⊗ denotes channel-wise multiplication with broadcasting and X is the original input feature map.

Overall, ECA couples global channel statistics with local inter-channel dependency modeling via neighborhood convolution, providing a lightweight yet effective attention mechanism. Compared with conventional channel-attention structures, ECA reduces computational complexity while maintaining strong representational ability.

## 3. Proposed Method

**Figure 4:**
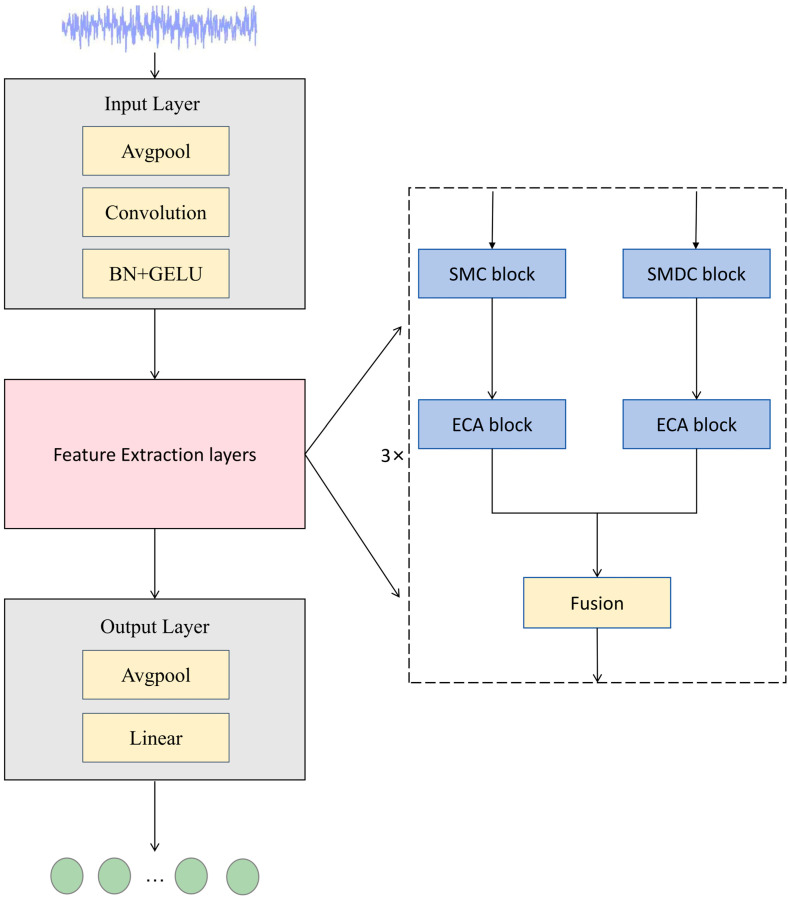
Overall architecture of the DSMC-ECA model.

This study proposes a lightweight fault diagnosis model for one-dimensional vibration signals, termed DSMC-ECA. As illustrated in Figure 4, the model consists of three components—an input module, a feature extraction module, and a classification module—enabling an end-to-end mapping from raw vibration signals to fault categories.

First, the raw vibration signal is fed into the input module, where average pooling and a convolutional block perform preliminary feature extraction and dimensional mapping, projecting the signal into a higher-dimensional feature space to obtain initial representations. The features are then passed to the feature extraction module for deep representation learning. This module adopts a dual-branch parallel architecture composed of a separable multi-scale convolution (SMC) branch and a separable multi-scale dilated convolution (SMDC) branch, which are designed with different emphases: the former focuses on local structure modeling, whereas the latter targets long-range dependency modeling. Through this explicit functional division, the two branches complement each other in extracting discriminative information.

Specifically, the SMC branch processes the input features in parallel using convolution kernels of different scales, emphasizing local structural patterns within a limited receptive field. This design is beneficial for capturing subtle impulsive signatures and short-range correlations in vibration signals. In contrast, the SMDC branch introduces dilation into the depthwise convolution stage and assigns different dilation rates to effectively enlarge the receptive field without a noticeable increase in parameters, thereby enhancing the modeling of cross-period modulation and long-range correlations. To further improve feature quality, an Efficient Channel Attention (ECA) module is applied in both branches for channel-wise recalibration. ECA extracts channel statistics via global average pooling and models local cross-channel dependencies using a one-dimensional convolution, which strengthens informative channel responses and suppresses redundant features and noise interference while keeping the computation lightweight. The outputs of the two branches are then fused, enabling the model to jointly exploit local details and global contextual representations, thus improving its capability to describe non-stationary and multi-scale vibration patterns. To further enrich hierarchical representations, the above dual-branch feature extraction module is stacked three times in the network, progressively refining more discriminative high-level features.

Finally, adaptive average pooling and a fully connected layer are employed to map the extracted high-dimensional features to the health-state space, yielding the final classification outputs for different fault categories.

The detailed structural parameters of the proposed DSMC-ECA model are summarized in Table 1.

## 4. Experimental Validation

### 4.1. Case 1: Xi’an Jiaotong University Planetary Gearbox Dataset

#### 4.1.1. Dataset Description

**Figure 5:**
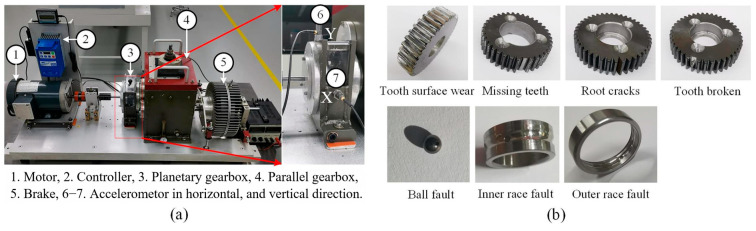
XJTU dataset: (**a**) experimental setup; (**b**) fault categories.

Case Study 1 is conducted on the public planetary gearbox dataset released by Xi’an Jiaotong University [44]. As shown in Figure 5a, the experimental platform mounts two accelerometers in the X and Y directions of the planetary gearbox to collect vibration signals. During data acquisition, the motor speed is fixed at 1800 r/min, and the sampling frequency is 20,480 Hz.

As illustrated in Figure 5b, the dataset includes nine health conditions in total: one normal state, four gear-related fault types, and four bearing-related fault types, among which one class corresponds to a compound (mixed) fault formed by three bearing fault modes. The diversity of fault categories makes this dataset representative and sufficiently challenging, which is well suited for evaluating fault diagnosis performance under complex operating conditions.

Following the signal segmentation principle, training samples are generated from the raw vibration signals of each health condition using a sliding-window strategy. Each sample contains 1024 sampling points, and 1200 samples are extracted for each condition, resulting in 10,800 samples in total. The dataset is split into the training, validation, and test sets with a ratio of 5:3:4. To avoid data leakage, no overlap is allowed between adjacent windows.

To further examine robustness under strong noise, additive Gaussian white noise is injected into the raw signals with different signal-to-noise ratios (SNR = −6 to 6 dB). The detailed sample allocation is summarized in Table 2.

#### 4.1.2. Experimental Setup and Result Analysis

All experiments were conducted under the same hardware and software environment to ensure a fair comparison. The experiments were carried out on a workstation running Windows 11, equipped with an Intel^®^ Core™ i7-12650H (2.30 GHz) CPU and an NVIDIA RTX 4060 (8 GB) GPU. The implementation was developed in Python 3.7.16 using PyTorch 1.11.0.

To comprehensively evaluate the proposed model, several representative CNN-based methods were selected for comparison, including MobileNet [32], MobileNetV2 [45], ResNet18 [46], MK-ResCNN [47], and the lightweight Transformer–CNN hybrid model LiConvFormer [38], CLFormer [48] and MCSAT [49]. All models were trained using the same data split and training strategy. To reduce the influence of randomness, each model was trained five times, and the average results were reported as the final performance. The batch size was set to 32 and the number of training epochs to 100. An initial learning rate of 0.001 was used for MCSAT, while 0.01 was used for the other models, with an adaptive learning-rate decay applied during training. Diagnostic accuracy and loss on both the training and validation sets were recorded throughout the process.

**Figure 6:**
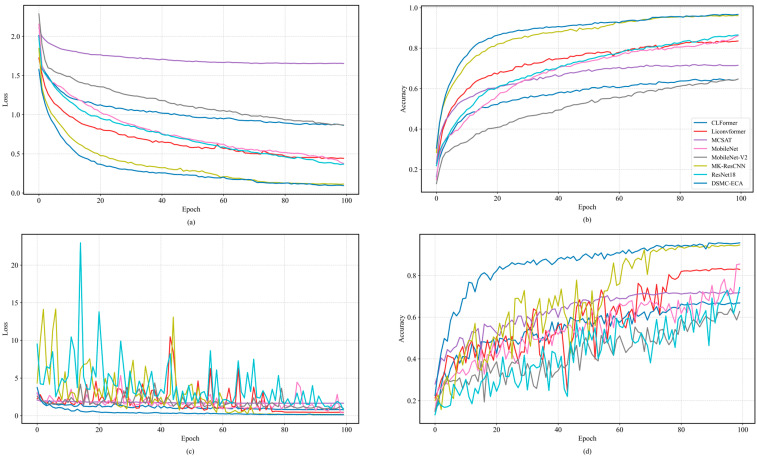
Loss and accuracy curves of different models: (**a**,**b**) training phase; (**c**,**d**) validation phase.

Figure 6 shows the loss and accuracy curves of different models on the planetary gearbox dataset under a severe noise condition (SNR = −6 dB) for both the training and validation phases. Clear performance differences can be observed among the compared methods. Overall, all models exhibit a relatively fast convergence trend during training; however, their stability and generalization ability in the validation phase vary considerably. As training proceeds, DSMC-ECA consistently achieves lower loss and higher diagnostic accuracy on both the training and validation sets, with a smoother convergence trajectory, indicating strong training stability and generalization under severe noise interference.

In comparison, MK-ResCNN achieves relatively high accuracy, but its validation curves exhibit considerable fluctuations, indicating that the model is sensitive to noise. LiConvFormer and MCSAT strike a certain balance between lightweight design and diagnostic performance, showing relatively stable overall performance; however, their final diagnostic accuracy is still lower than that of DSMC-ECA, especially in the later stages of training. The validation accuracy of CLFormer is much lower than that of the other models, which can be attributed to its feature dimensionality reduction strategy that results in the loss of detailed information in the vibration signals. These experimental results demonstrate the effectiveness and robustness of the proposed DSMC-ECA for gearbox fault diagnosis under complex noisy conditions.

Table 3 compares the diagnostic accuracy, computational complexity, and parameter count of different models on the XJTU planetary gearbox dataset under multiple signal-to-noise ratio (SNR) settings. Across a wide range of operating conditions—from −6 dB to 6 dB, as well as the noise-free case—the proposed DSMC-ECA consistently achieves superior overall accuracy, demonstrating strong robustness to noise.

As the SNR decreases, the diagnostic performance of all models degrades to varying degrees; nevertheless, the advantage of DSMC-ECA becomes more pronounced under severe noise. Under the most challenging condition (SNR = −6 dB), DSMC-ECA still attains an accuracy of 95.11%, outperforming the second-best method MK-ResCNN by 0.69%. This result indicates that DSMC-ECA maintains more stable feature extraction and discrimination capability when fault-relevant signatures are strongly masked by noise.

Moreover, DSMC-ECA achieves high diagnostic accuracy while maintaining relatively low model complexity, with only 0.204 M parameters and a computational cost of 10.037 M FLOPs, striking a favorable balance between accuracy and efficiency. In contrast, lightweight models such as CLFormer and MCSAT, despite having fewer parameters, see their accuracy drop to 67.01% and 72.04% under high-noise conditions, indicating insufficient capability in modeling fine-grained discriminative features in complex vibration signals. Other baseline models, such as MobileNet and ResNet18, not only involve higher computational complexity but also exhibit lower diagnostic accuracy than DSMC-ECA under low signal-to-noise ratio conditions.

**Figure 7:**
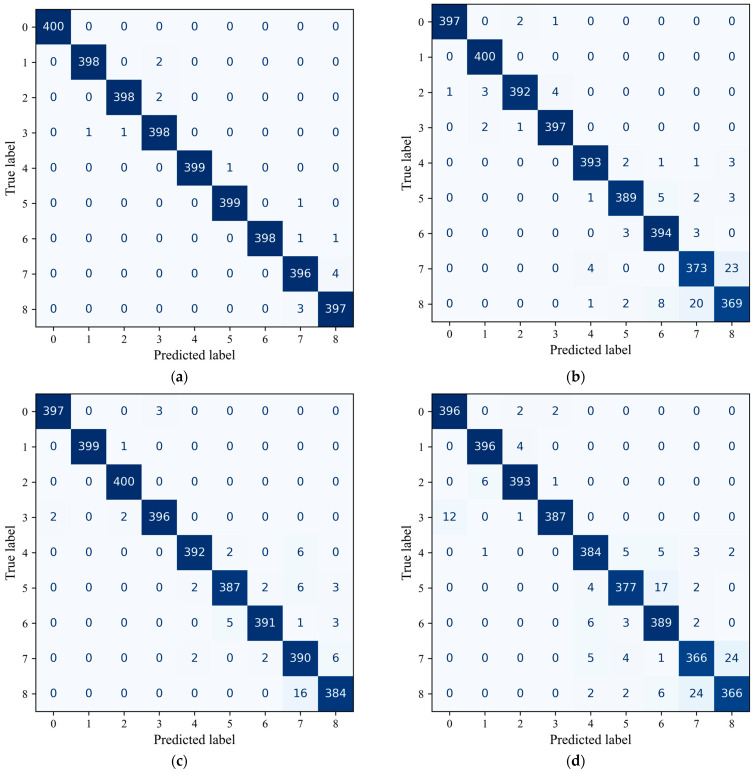

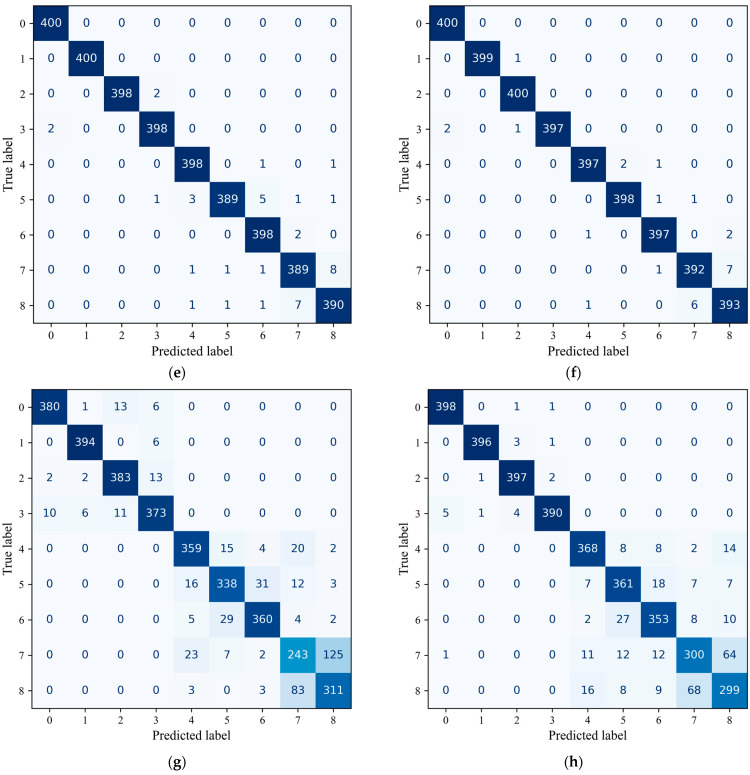
Confusion matrices of different models on the XJTU dataset. (**a**) DSMC-ECA; (**b**) Liconvformer; (**c**) MobileNet; (**d**) MobileNet-V2; (**e**) ResNet18; (**f**) MK-ResCNN; (**g**) CLFormer; (**h**) MCSAT.

To further assess classification behavior under noise, confusion matrices of the six models at SNR = 0 dB are reported in Figure 7, where the horizontal and vertical axes (0–8) correspond to the nine health conditions listed in Table 2.

As shown in Figure 7, DSMC-ECA correctly recognizes almost all health conditions, with only a very small number of misclassifications between Class 7 (planetary rootcracks) and Class 8 (planetary toothwear). This confusion is mainly attributed to the similarity of their vibration responses; under strong noise, the subtle differences between these two fault patterns become further weakened, increasing the difficulty of discrimination.

By comparison, MK-ResCNN and ResNet18 yield competitive overall performance, yet still produce noticeable misclassifications for some categories, especially among fault types with similar signatures. The remaining baselines exhibit more severe confusion across multiple categories. In particular, MobileNetV2 shows a relatively dispersed prediction distribution over many classes, implying limited capability in modeling complex vibration patterns and suppressing noise.

The poorest performance is observed in the two most lightweight models, CLFormer and MCSAT, which exhibit a large number of misclassifications in the more challenging classes 7 and 8. This indicates that when confronted with faults having highly similar vibration characteristics, the discriminative capability of these two models is relatively limited.

Overall, the confusion-matrix results confirm that DSMC-ECA provides more stable and robust classification under noise, enabling clearer discrimination among different fault types.

To comprehensively evaluate the classification performance of each model under varying noise conditions, this study introduces the F1-score and recall metrics to complement the limitations of relying solely on accuracy. Table 4 presents the mean recall and F1-score of each model on the XJTU dataset across different SNR levels (−6 dB to 0 dB). The results indicate that DSMC-ECA consistently outperforms other models under all SNR conditions. Even under the most severe noise scenario (SNR = −6 dB), it achieves a mean recall of 95.53% and an F1-score of 95.52%, demonstrating not only high overall accuracy but also strong class discriminability and robustness to noise. The second-best model, MK-ResCNN, also maintains relatively high performance across different noise levels, although its metrics noticeably decline as noise increases. In contrast, lightweight models such as CLFormer and MCSAT, despite having the smallest parameter counts, exhibit the most significant performance degradation under high-noise conditions, with F1-scores of only 66.46% and 71.83% at SNR = −6 dB, reflecting their limited capability in capturing fine-grained discriminative features from complex vibration signals. Overall, these results further validate that DSMC-ECA can maintain high performance under increasing noise levels, highlighting its superiority in both noise robustness and fault diagnosis accuracy.

**Figure 8:**
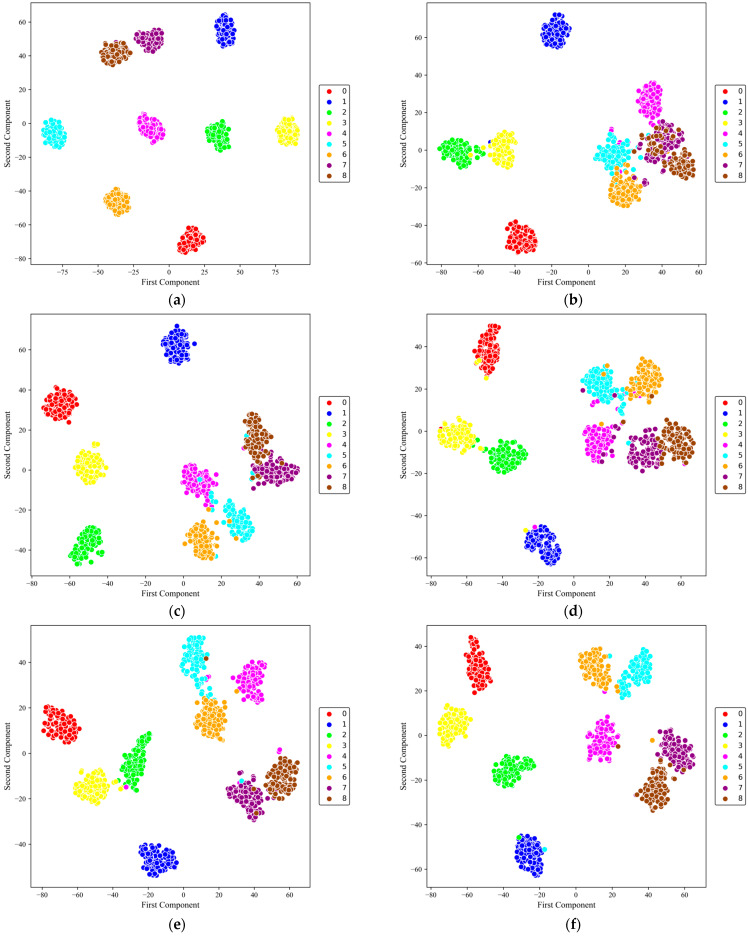

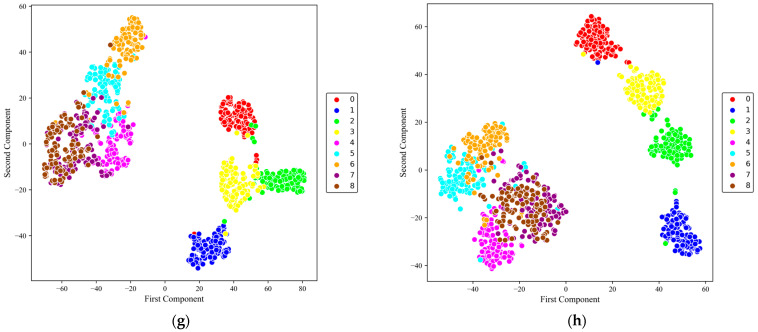
t-SNE visualization on the XJTU dataset at SNR = 0 dB. (**a**) DSMC-ECA; (**b**) Liconvformer; (**c**) MobileNet; (**d**) MobileNet-V2; (**e**) ResNet18; (**f**) MK-ResCNN; (**g**) CLFormer; (**h**) MCSAT.

Figure 8 presents the t-SNE visualization of the learned feature representations (from the fully connected layer output) for the six models under SNR = 0 dB. This visualization helps assess the compactness within classes and the separability between them in the learned feature space.

From the overall distribution, the models show substantially different clustering behavior. DSMC-ECA forms compact intra-class clusters with relatively clear boundaries between most health conditions, and only slight overlap is observed between a few similar fault categories. This suggests that the proposed dual-branch multi-scale feature extractor, together with channel attention, strengthens discriminative representation learning under noise.

In contrast, MK-ResCNN shows the second-best separability: most classes remain clustered, but blurred boundaries are still visible for certain categories. ResNet18 and other baselines present more pronounced inter-class mixing, with substantial overlap among similar fault types, indicating limited ability to capture fine-grained discriminative differences under noisy conditions. The two lowest-performing models, CLFormer and MCSAT, show pronounced class overlap.

### 4.2. Case 2: Southeast University Gearbox Dataset

#### 4.2.1. Dataset Description

**Figure 9:**
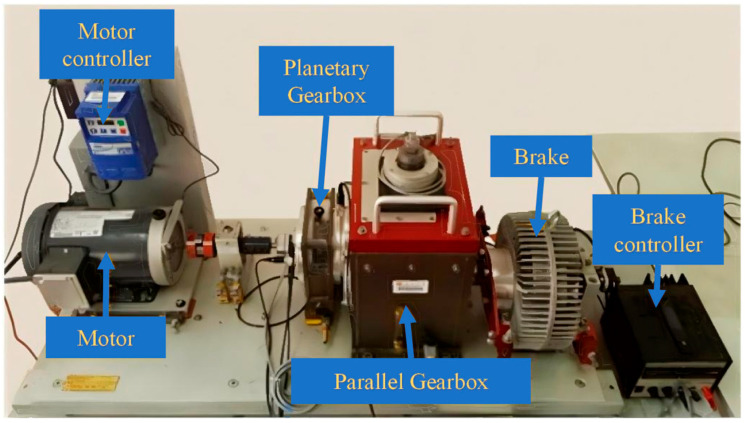
Data acquisition platform for the SEU dataset.

Case Study 2 is conducted on the public Southeast University gearbox dataset [50]. As shown in Figure 9, the experimental platform is built upon a drivetrain simulator and consists of a motor, motor controller, planetary gearbox, parallel gearbox, brake, and brake controller. This test rig enables the acquisition of multi-source operational data under different working conditions, providing a solid experimental basis for fault diagnosis research in rotating machinery.

The SEU dataset provides vibration signals measured along the x-, y-, and z-axes for both the parallel gearbox and the planetary gearbox, and also includes motor vibration signals and torque data. In total, vibration data are collected under ten health conditions, covering four bearing fault types, four gear fault types, one healthy bearing condition, and one healthy gear condition. Owing to the rich fault categories and complex operating settings, this dataset is representative and challenging for evaluating diagnostic models.

In this study, we use the data recorded under the load condition of 20 Hz–0 V, and select the x-axis vibration signal of the planetary gearbox as the model input. To ensure consistency and comparability across experiments, the raw vibration signals are preprocessed in a unified manner. First, the signals are segmented using a non-overlapping sliding window, where each sample contains 1024 sampling points. Then, the dataset is split into training, validation, and test sets with a ratio of 60%/20%/20%.

To further assess the robustness of the proposed DSMC-ECA under noise contamination, Gaussian white noise is added to the original vibration signals under different signal-to-noise ratios (SNR = −6 to 6 dB). The number of samples and label distribution after preprocessing are summarized in Table 5.

#### 4.2.2. Experimental Results and Discussion

Table 6 reports the diagnostic accuracy and model complexity of different methods on the SEU gearbox dataset under multiple SNR settings. As can be observed, across a wide range of conditions from SNR = −6 dB to the noise-free case, DSMC-ECA achieves the best or near-best performance, indicating strong adaptability and stability under varying noise levels and operating conditions.

Under severe noise (SNR = −6 dB), the diagnostic performance of all baseline models degrades to different extents. In particular, lightweight models such as MobileNetV2 and LiConvFormer exhibit a more pronounced accuracy drop, suggesting limited capability in capturing discriminative features when fault signatures are heavily masked by noise. In contrast, DSMC-ECA still maintains a relatively high accuracy at −6 dB, demonstrating that the proposed dual-branch multi-scale separable convolution design can extract informative temporal features even under strong noise contamination.

From the perspective of computational efficiency, DSMC-ECA achieves competitive accuracy while requiring significantly fewer parameters and lower computational cost than mainstream CNN baselines such as MobileNet, MobileNetV2, and ResNet18. Although CLFormer and MCSAT have the lowest parameter count and computational complexity, their diagnostic accuracy is also the lowest, reflecting that their ability to extract discriminative features from complex vibration signals is limited despite being lightweight. These results confirm that the proposed integration of multi-scale separable convolution and efficient channel attention provides a favorable trade-off between diagnostic performance and computational overhead on the SEU dataset.

**Figure 10:**
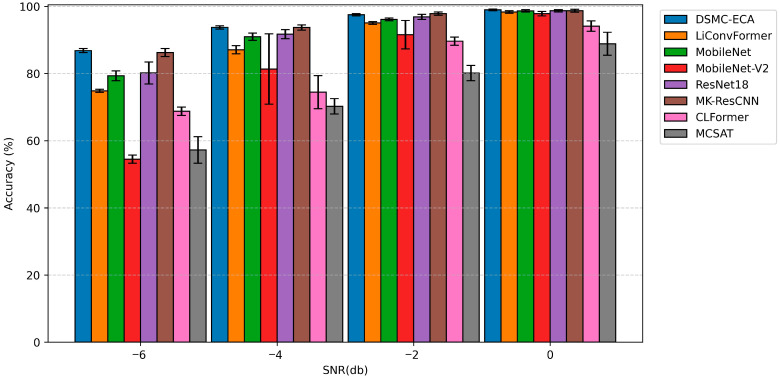
Mean diagnostic accuracy of different models on the SEU dataset under various SNR conditions.

To provide a more intuitive comparison of robustness under strong noise, Figure 10 presents bar-chart results of diagnostic accuracy on the SEU dataset at SNR = −6, −4, −2, and 0 dB, where error bars indicate the performance variation across repeated trials. Since most models tend to saturate at medium-to-high SNRs (making differences difficult to distinguish), this study focuses on the high-noise regime for visualization, while the full results for other SNR levels are summarized in Table 6.

As shown in Figure 10, DSMC-ECA achieves a higher mean diagnostic accuracy than the other comparative methods under all high-noise conditions, while exhibiting overall smaller error bars. This indicates that the proposed model delivers more stable performance across repeated trials and thus provides more consistent and comparable results. In particular, under the most challenging condition of SNR = −6 dB, although the diagnostic accuracy of all models decreases to some extent, DSMC-ECA still maintains the highest average accuracy. Moreover, its error range is smaller than that of most competing methods, demonstrating that DSMC-ECA is better suited for feature learning in severely noisy environments.

With the gradual increase in SNR, the accuracy gaps among different models shrink and tend to saturate at around 0 dB, and the error bars generally become shorter. This suggests that model training is more stable in low-noise settings and that the impact of network architecture on diagnostic performance is relatively weakened. By contrast, performance differences become more pronounced under strong noise interference, further highlighting that multi-scale feature extraction and the efficient channel attention mechanism play a crucial role in suppressing noise and improving feature stability.

**Figure 11:**
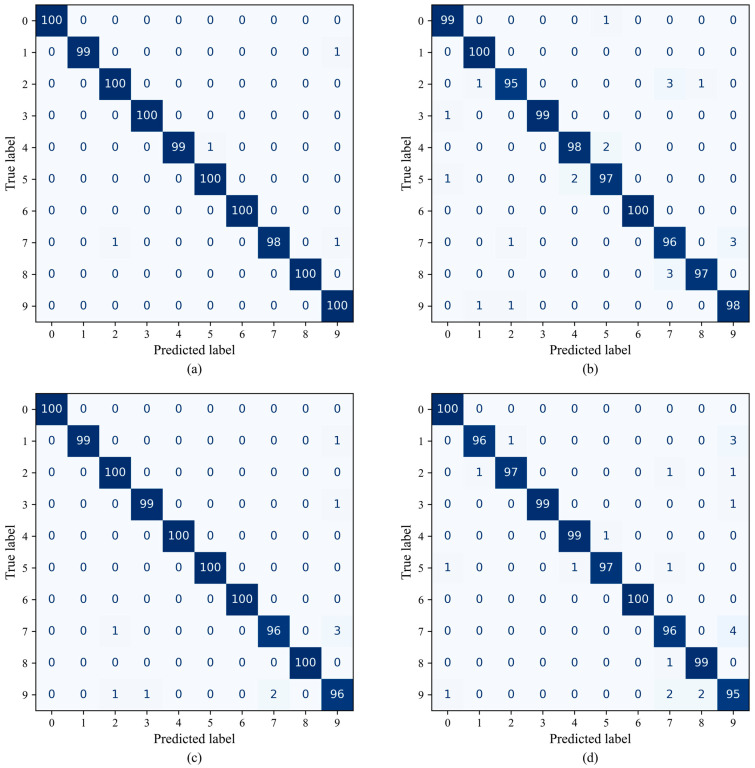

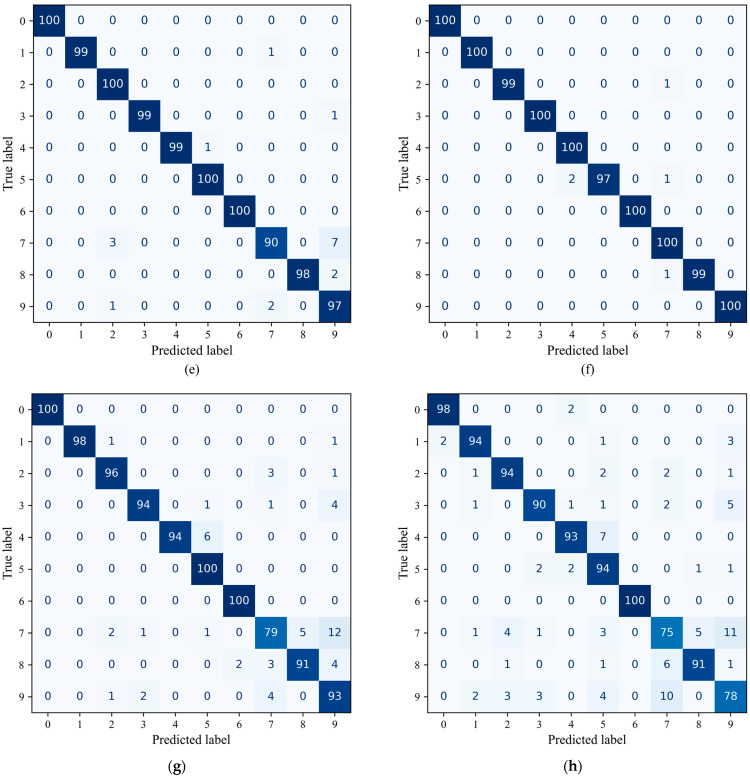
Confusion matrices of different models on the SEU dataset. (**a**) DSMC-ECA; (**b**) Liconvformer; (**c**) MobileNet; (**d**) MobileNet-V2; (**e**) ResNet18; (**f**) MK-ResCNN; (**g**) CLFormer; (**h**) MCSAT.

To further analyze the classification performance of different models, Figure 11 presents the confusion matrices under SNR = 0 dB. As shown in Figure 11, DSMC-ECA achieves high recognition accuracy for both bearing-related and gear-related faults on the SEU dataset, with only a few misclassifications. This indicates that the proposed method can maintain stable discriminative capability even when confronted with more diverse fault categories.

In contrast, the other compared models exhibit varying degrees of confusion among multiple fault classes, suggesting that their feature extraction and discrimination abilities remain limited under complex operating conditions and noise interference.

Table 7 reports the mean recall and F1-score of different models on the SEU dataset under various SNR conditions ranging from −6 dB to 0 dB. The overall trends are consistent with those observed in the first experiment. DSMC-ECA demonstrates superior noise robustness compared with other baseline models, particularly under high-noise conditions. Although its mean recall and F1-score are slightly lower than those of MK-ResCNN under some moderate noise levels, DSMC-ECA achieves the best performance under the most severe noise condition (SNR = −6 dB). The stable performance across different noise levels further indicates that DSMC-ECA exhibits the best overall diagnostic capability in terms of robustness and classification effectiveness.

**Figure 12:**
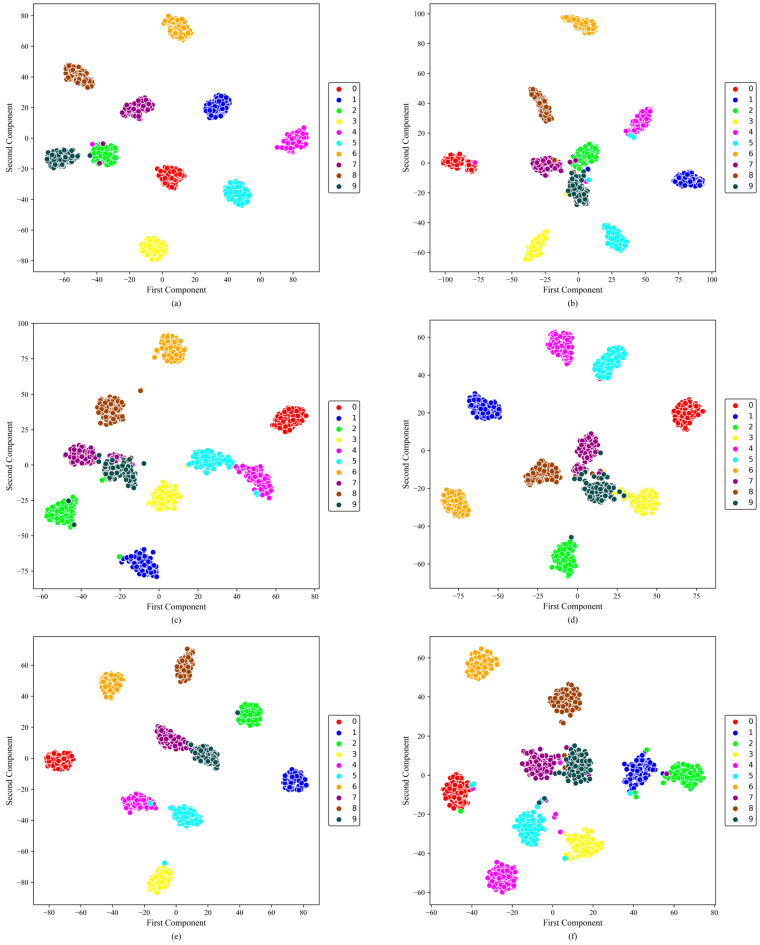

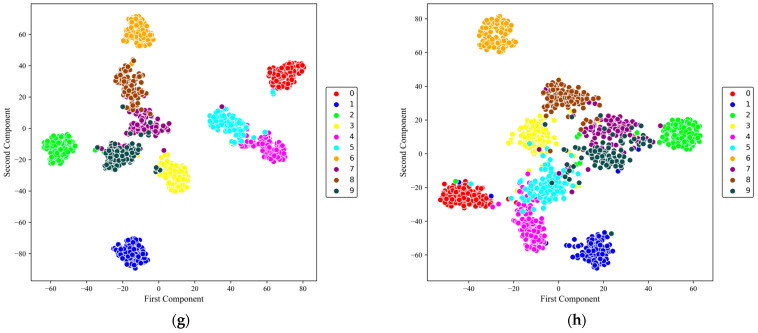
t-SNE visualization on the SEU dataset at SNR = 0 dB. (**a**) DSMC-ECA; (**b**) Liconvformer; (**c**) MobileNet; (**d**) MobileNet-V2; (**e**) ResNet18; (**f**) MK-ResCNN; (**g**) CLFormer; (**h**) MCSAT.

In addition, Figure 12 illustrates the two-dimensional distributions of the features extracted from the fully connected layer of each model using t-SNE under the 0 dB noise condition. In this setting, the learned features of all models are generally distinguishable. However, DSMC-ECA shows a clearer separation among different fault categories, with more compact intra-class clusters and more distinct inter-class boundaries. These results indicate that the proposed method can learn highly discriminative feature representations. In contrast, the other models exhibit more severe inter-class overlap, suggesting that their modeling capability under complex fault conditions still needs improvement.

### 4.3. Ablation Experiment

**Figure 13:**
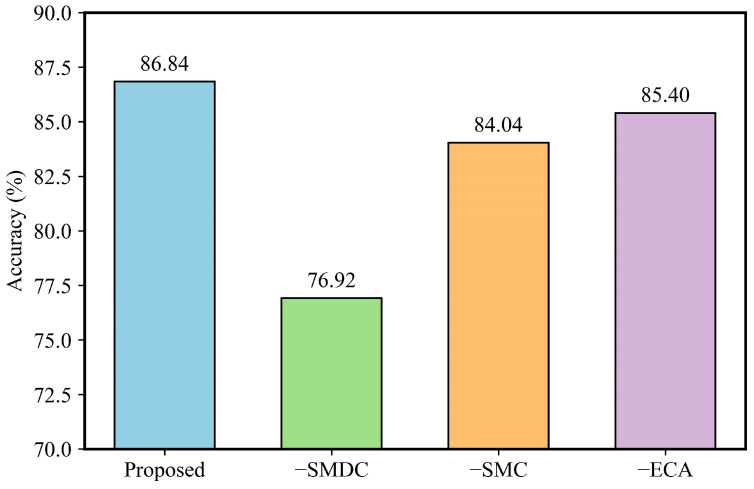
Comparison of diagnostic accuracy for different ablation settings.

Ablation experiments are performed on the SEU gearbox dataset to quantify the impact of each key component in the proposed model. Starting from the complete DSMC-ECA architecture, we separately remove the separable multi-scale convolution module (SMC), the separable multi-scale dilated convolution module (SMDC), and the efficient channel attention module (ECA) to construct three variant models. All variants are trained under the same hyperparameter configuration to ensure a fair comparison. In particular, all ablation experiments are performed under a severe noise condition (SNR = −6 dB) to evaluate the role of each module in challenging environments. The results are summarized in Figure 13 and Table 8.

As shown in Table 6, the complete DSMC-ECA model achieves the highest diagnostic accuracy under the severe-noise condition (SNR = −6 dB), reaching 86.84%, while maintaining a low computational cost of 10.037 M FLOPs and only 0.204 M parameters. This demonstrates that the proposed module design can achieve a favorable balance between accuracy and efficiency in strong-noise environments. When the SMDC branch is removed, the accuracy drops markedly to 76.92% (a decrease of 9.92%), although the computational complexity and parameter count are reduced to 5.051 M FLOPs and 0.103 M, respectively. Similarly, removing the SMC branch decreases the accuracy to 84.04% (a decrease of 2.80%), with a complexity of 5.125 M FLOPs and 0.105 M parameters. These results indicate that both the SMC and SMDC branches contribute to performance improvement. In contrast, removing ECA only causes a minor accuracy reduction to 85.40% (a decrease of 1.44%), while the model complexity and parameter count remain almost unchanged (9.911 M FLOPs, 0.204 M), suggesting that ECA primarily improves feature quality through channel-wise recalibration without introducing noticeable additional computational burden.

Further comparison shows that, under severe noise, the SMDC module has the most significant impact on accuracy. This is because SMDC introduces a dilation mechanism on top of the SMC framework, effectively enlarging the receptive field with a limited increase in model cost. As a result, it enhances the ability to model long-range dependencies and cross-scale correlations in vibration signals, allowing the model to retain strong discriminative representations even when fault features are heavily masked by noise. This further confirms the effectiveness of the proposed improvement. By comparison, SMC mainly focuses on capturing local structural patterns and fine-grained impulsive features, and its removal weakens the representation of critical fault details. ECA, meanwhile, adaptively reweights channel responses to emphasize informative channels and suppress noise-dominant redundancy, providing a stable yet lightweight performance gain.

In summary, SMC, SMDC, and ECA play complementary roles in DSMC-ECA, namely local feature extraction, long-range multi-scale dependency modeling, and channel-wise feature selection. Their integration enables the proposed model to jointly exploit local details and global contextual information, leading to significantly improved gearbox fault diagnosis performance under severe noise while maintaining low computational overhead. These ablation results validate the rationality and effectiveness of the proposed network design.

## 5. Conclusions

This paper proposes a lightweight gearbox fault diagnosis method, termed DSMC-ECA, based on improved multi-scale separable convolution and efficient channel attention (ECA). The proposed method employs a dual-branch feature extraction architecture: the SMC branch focuses on modeling local fine-grained impulsive information, while the SMDC branch enhances the receptive field through dilated separable convolutions to capture cross-scale correlations and long-range dependency features. Meanwhile, ECA is introduced to perform channel-wise adaptive recalibration of multi-scale features, which highlights critical fault-related information and suppresses noise interference with negligible additional computational overhead. Through feature fusion, the proposed framework enables the sharing and complementarity between fine-grained details and global multi-scale representations.

Experimental results on the XJTU and SEU datasets demonstrate that DSMC-ECA achieves stable and superior diagnostic performance across a wide range of signal-to-noise ratios (from −6 dB to noise-free conditions), consistently outperforming multiple baseline models. In particular, under the most challenging setting of SNR = −6 dB, DSMC-ECA attains the highest average diagnostic accuracies of 95.11% (XJTU) and 86.84% (SEU), improving upon the second-best MK-ResCNN by 0.69% and 0.60%, respectively. Moreover, ablation studies on the SEU dataset further verify the contributions of each module to noise robustness and their synergistic gains. In terms of model complexity, DSMC-ECA contains only 0.204 M parameters and requires 10.037 M FLOPs, achieving a favorable balance between diagnostic performance and computational efficiency, and thus showing strong potential for deployment in resource-constrained industrial environments and edge-computing scenarios.

Nevertheless, several limitations of the proposed DSMC-ECA method should be acknowledged. First, although the experimental results under multiple noise levels sufficiently demonstrate the noise robustness of the proposed method, the noise conditions considered in this study are mainly artificially added and relatively controlled. In real industrial environments, noise sources are often more complex and exhibit strong non-stationary characteristics, which may adversely affect the feature extraction process. In addition, the proposed method is mainly evaluated under fixed-speed and fixed-load conditions, and its adaptability to practical engineering scenarios involving variable-speed and variable-load operating conditions has not yet been systematically investigated.

Future work can be further extended in the following directions:

(1) Incorporating more rotating machinery datasets and real industrial operating conditions to further validate the model’s performance in practical applications;
(2) Investigating different signal representations and multimodal fusion strategies to enable collaborative modeling and diagnosis of multi-source heterogeneous vibration signals;
(3) Further improving computational efficiency to enhance the method’s applicability to real-time online monitoring and industrial edge computing.

## Figures and Tables

**Table 1:** Structural configuration of the proposed DSMC-ECA model.

Layer	Blocks	Parameters	Signal Shape
Input signal	–	–	(B, n, 1024)
Input layer	Average pooling	k = 2; s = 2	(B, n, 512)
	Convolution block	k = 15; s = 2	(B, 32, 256)
Feature extraction layer 1	SMC block	k = 3, 5, 7, 9; s = 2	(B, 64, 128)
	SMDC block	k = 3; d = 2, 4, 8, 16; s = 2	(B, 64, 128)
	ECA block	adaptive k	(B, 64, 128)
Feature extraction layer 1	SMC block	k = 3, 5, 7, 9; s = 2	(B, 128, 64)
	SMDC block	k = 3; d = 2, 4, 8, 16; s = 2	(B, 128, 64)
	ECA block	adaptive k	(B, 128, 64)
Feature extraction layer 1	SMC block	k = 3, 5, 7, 9; s = 2	(B, 256, 32)
	SMDC block	k = 3; d = 2, 4, 8, 16; s = 2	(B, 256, 32)
	ECA block	adaptive k	(B, 256, 32)
Output layer	Average pooling	k = 32; s = 32	(B, 256)
	Linear	–	(B, C)

The input signal length is 1024 sampling points, and B denotes the batch size, k denotes the kernel size, s denotes the stride, d denotes the dilation rate in dilated convolutions, and C denotes the number of healthy states. For the ECA module, the 1D convolution kernel size k is adaptively determined by the channel number.

**Table 2:** Sample split of the XJTU dataset.

Type of Fault	Train Data	Valid Data	Test Data	Fault Category
Bearing Ball	500	300	400	0
Bearing Inner	500	300	400	1
Bearing Mix	500	300	400	2
Bearing Outer	500	300	400	3
Planetary Brokentooth	500	300	400	4
Planetary Missingtooth	500	300	400	5
Normal	500	300	400	6
Planetary Rootcracks	500	300	400	7
Planetary Toothwear	500	300	400	8

**Table 3:** Accuracy and model complexity comparisons on the XJTU dataset.

Methods	Diagnostic Accuracy (%)								Complexity (M)	
	XJTU Dataset SNR (dB)									
	−6	−4	−2	0	2	4	6	None	Flops	Parameters
DSMC-ECA	95.11	97.92	99.31	99.60	99.78	99.75	99.84	99.89	10.037	0.204
Liconvformer	83.67	92.23	95.74	97.81	98.72	98.86	99.22	99.41	14.520	0.323
MobileNet	85.85	94.27	97.21	98.57	98.98	98.99	99.07	99.67	333.620	3.186
MobileNet-V2	65.01	79.68	93.69	96.37	98.16	98.12	98.39	99.66	96.955	2.192
ResNet18	85.51	94.62	98.56	98.94	99.23	99.38	99.37	99.66	175.920	3.854
MK-ResCNN	94.42	97.87	98.99	99.30	99.68	99.60	99.77	99.70	83.893	2.117
CLformer	67.01	72.43	79.62	86.14	87.88	90.02	91.57	92.08	0.005	0.143
MCSAT	72.04	80.95	87.74	90.80	94.47	96.56	97.59	99.11	8.587	0.044

**Table 4:** Model Performance on the XJTU dataset:mean recall and mean F1-score.

Methods	XJTU Dataset SNR (dB)	Mean Recall (%)	Mean F1-Score (%)
DSMC-ECA	−6	95.53	95.52
	−4	98.14	98.14
	−2	99.32	99.32
	0	99.51	99.51
Liconvformer	−6	83.43	83.38
	−4	91.17	91.15
	−2	96.76	96.76
	0	98.02	98.02
MobileNet	−6	85.61	85.52
	−4	94.40	94.40
	−2	97.47	97.47
	0	98.49	98.49
MobileNet-V2	−6	65.54	65.30
	−4	81.06	81.01
	−2	90.19	90.11
	0	96.63	96.73
ResNet18	−6	85.51	85.11
	−4	94.66	94.60
	−2	98.32	98.32
	0	98.91	98.91
MK-ResCNN	−6	94.46	94.46
	−4	97.74	97.74
	−2	98.83	98.83
	0	99.52	99.52
CLFormer	−6	67.01	66.46
	−4	72.43	72.15
	−2	79.62	79.47
	0	86.14	86.07
MCSAT	−6	72.04	71.83
	−4	80.95	80.84
	−2	87.74	87.74
	0	90.80	90.78

**Table 5:** Sample split of the SEU dataset.

Type of Fault	Train Data	Valid Data	Test Data	Fault Category
Bearing Ball	300	100	100	0
Bearing Comb	300	100	100	1
Bearing Health	300	100	100	2
Bearing Inner	300	100	100	3
Bearing Outer	300	100	100	4
Gearset Chipped	300	100	100	5
Gearset Health	300	100	100	6
Gearset Miss	300	100	100	7
Gearset Root	300	100	100	8
Gearset Surface	300	100	100	9

**Table 6:** Accuracy and model complexity comparisons on the SEU dataset.

Methods	Diagnostic Accuracy (%)								Complexity (M)	
	SEU Dataset SNR (dB)									
	−6	−4	−2	0	2	4	6	None	Flops	Parameters
DSMC-ECA	86.84	93.97	97.50	98.96	99.66	99.68	99.80	99.98	10.037	0.204
Liconvformer	74.84	87.08	95.06	98.32	99.14	99.52	99.56	99.58	14.520	0.323
MobileNet	76.04	90.96	96.12	98.66	99.20	99.36	99.56	99.56	333.620	3.186
MobileNet-V2	54.50	81.34	91.58	97.84	99.04	99.52	99.52	99.64	96.955	2.192
ResNet18	80.16	91.74	96.88	98.68	99.38	99.58	99.66	99.70	175.920	3.854
MK-ResCNN	86.24	93.70	97.84	98.70	99.44	99.54	99.58	99.72	83.893	2.117
CLFormer	68.76	74.46	89.62	94.12	97.64	98.68	99.30	99.78	0.005	0.133
MCSAT	57.24	70.24	80.14	88.86	94.58	97.00	96.06	97.40	8.497	0.044

**Table 7:** Model Performance on the SEU dataset: mean recall and mean F1-score.

Methods	SEU Dataset SNR (dB)	Mean Recall (%)	Mean F1-Score (%)
DSMC-ECA	−6	86.12	86.18
	−4	93.60	93.61
	−2	97.50	97.51
	0	98.96	98.96
Liconvformer	−6	75.66	75.35
	−4	87.94	87.93
	−2	95.22	95.24
	0	98.48	98.48
MobileNet	−6	77.60	77.53
	−4	91.30	91.32
	−2	96.38	96.39
	0	98.62	98.62
MobileNet-V2	−6	58.78	58.45
	−4	78.22	78.15
	−2	94.28	94.29
	0	98.40	98.40
ResNet18	−6	83.92	83.88
	−4	92.18	92.19
	−2	97.66	97.67
	0	98.86	98.86
MK-ResCNN	−6	85.76	85.81
	−4	94.52	94.53
	−2	97.34	97.34
	0	99.02	99.02
CLformer	−6	68.76	67.74
	−4	74.46	73.89
	−2	89.62	89.63
	0	94.12	94.15
MCSAT	−6	57.24	56.32
	−4	70.24	69.99
	−2	80.14	80.10
	0	88.86	88.15

**Table 8:** Ablation results on the SEU dataset (SNR = −6 dB).

Methods	Diagnostic Accuracy (%)	Complexity (M)	
		Flops	Parameters
Proposed	86.84	10.037	0.204
−SMDC	76.92	5.125	0.105
−SMC	84.04	5.051	0.103
−ECA	85.40	9.911	0.204

## Data Availability

The dataset used in this article can be obtained from the corresponding author upon request.
